# Home blood pressure control and prescribing patterns of anti-hypertensive medications in a home blood pressure-based hypertension-specialized clinic in Japan: a sub-analysis of the Ohasama study

**DOI:** 10.1038/s41440-024-01954-7

**Published:** 2024-10-28

**Authors:** Michihiro Satoh, Hirohito Metoki, Takahisa Murakami, Yukako Tatsumi, Kei Asayama, Masahiro Kikuya, Takayoshi Ohkubo, Yutaka Imai

**Affiliations:** 1 Division of Public Health, Hygiene and Epidemiology, Faculty of Medicine, Tohoku Medical and Pharmaceutical University, Sendai, Japan; 2 Department of Preventive Medicine and Epidemiology, Tohoku Medical Megabank Organization, Tohoku University, Sendai, Japan; 3 Department of Pharmacy, Tohoku Medical and Pharmaceutical University Hospital, Sendai, Japan; 4 Tohoku Institute for Management of Blood Pressure, Sendai, Japan; 5 Division of Aging and Geriatric Dentistry, Department of Rehabilitation Dentistry, Tohoku University Graduate School of Dentistry, Sendai, Japan; 6 Department of Hygiene and Public Health, Teikyo University School of Medicine, Tokyo, Japan

**Keywords:** Home Blood Pressure, Blood Pressure Monitoring, Ambulatory, Cross-Sectional Studies, Epidemiology, Blood Pressure Control

## Abstract

Although the benefits of anti-hypertensive treatment are well known, the proportion of hypertensive patients with controlled blood pressure (BP) remains suboptimal. The present study aimed to compare BP control conditions in a hypertension-specialized clinic and non-hypertension-specialized clinics. This cross-sectional study used data from 379 treated patients who measured home BP in the Ohasama study between 2016 and 2019 (men: 43.0%, age: 71.6 years). Of those, 172 patients were managed at the hypertension-specialized clinic where physicians distributed home BP devices to each patient, evaluated the home BP data, and adjusted medications to maintain home BP values according to the recent Japanese guidelines. When we set morning home systolic/diastolic BP of <135/ < 85 mmHg as controlled BP, 93.6% of patients fulfilled the controlled home BP range, compared to 43.0% in non-specialized clinics (*n* = 207). The proportion of the patients with home morning BP < 125/ < 75 mmHg was 73.3% in the hypertension-specialized clinic and 20.8% in the non-hypertension-specialized clinics. Hypertension-specialized clinics prescribed three or more anti-hypertensive drug classes to 41.9% of patients, compared to 15.2% in non-specialized clinics. In the hypertension-specialized clinic, angiotensin II receptor blockers were most commonly prescribed (86.6%), followed by dihydropyridine calcium channel blockers (77.9%), thiazide (including thiazide-like) diuretics (30.2%), mineralocorticoid receptor blockers (23.8%), and beta- and alpha-beta blockers (10.5%). In conclusion, the proportion of patients with controlled home BP was excellent in the hypertension-specialized clinic. Home BP-based hypertension practices, as recommended in the current Japanese guidelines, may be the key to achieving sufficient BP control.

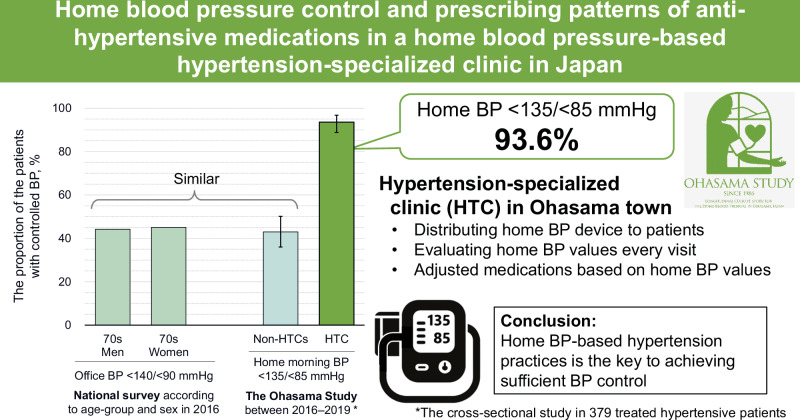

## Introduction

Hypertension is a common chronic disease worldwide with significant health implications [1–4]. Lowering blood pressure (BP) through intensive anti-hypertensive treatment is beneficial for patients with hypertension [5–7]. Despite accumulating evidence of the benefits of anti-hypertensive treatments, the proportion of hypertensive patients with controlled BP remains suboptimal [1, 8–12]. This is considered to be due to patients’ poor drug adherence and so-called “clinical inertia,” which is a state in which healthcare providers fail to provide initial treatment or increase treatment where required [13]. Poor hypertension control is associated with an increased risk of cardiovascular diseases and other adverse health outcomes [14, 15]. Therefore, healthcare professionals have developed several strategies to effectively manage this condition [1–3].

Japan was the first country to introduce self-measured BP at home (home BP) for the management of hypertension, and it has since been adopted in other countries [1–3]. A meta-analysis of randomized controlled trials reported that treatment based on home BP measurement lowered ambulatory BP levels in hypertensive patients undergoing anti-hypertensive measurements [16]. Moreover, home BP encourages active participation of patients in the management of hypertension [17]. At the hypertension-specialized clinic (HTC) in Ohasama town, we have treated outpatients based on continual home BP monitoring unless exceptional circumstances.

In the present study, we aim to assess BP control in the HTC by comparing BP control among outpatients with that in non-HTC outpatients, where hypertension is considered to be conventionally managed. We then identified factors that affect the management of hypertension, including those related to the clinical inertia.

Point of view

- Clinical relevance
  A stringent home blood pressure-based management at a hypertension-specialized clinic (HTC) can improve hypertension control rates.
- Future direction
  Exploring how to expand the HTC model and home blood pressure monitoring in Asian general practice is beneficial.
- Consideration for the Asian population
  Improving blood pressure control remains a challenge in Asia. Implementing strict management based on home blood pressure measurement as demonstrated in this study, may offer a solution to this persistent issue.

## Methods

### Study design and populations

This cross-sectional study was part of the Ohasama study, which was conducted in Ohasama town, Hanamaki city, Japan. The details of the study and the demographic characteristics of this region have been described previously [18**–**21].

The Ohasama study investigates one district each year and completes the surveys of entire districts of Ohasama town for 4 years. Therefore, we used 4 years of data collected, which were the most recent data available on anti-hypertensive treatment at the time of writing this report. This study adhered to the Declaration of Helsinki, and the Institutional Review Boards of Teikyo University (approval number: 16-075-8) and Tohoku Medical and Pharmaceutical University (approval number: 2016-0-001-0006 approved the study protocol.

Between 2016 and 2019, 865 patients participated in the Ohasama study, and 829 provided informed consent. Of all, 16 participants without home BP for ≥3 days and 434 who did not receive antihypertensive treatment were excluded. Finally, we included 379 patients undergoing antihypertensive treatment (men: 43.0%; age: 71.6 years) who participated in the Ohasama study and measured their home BP during the study period. Of these, 245 patients (men: 41.2%; age: 71.0 years) had office BP information during the study examination.

To assess the longitudinal home BP change including the pre-treatment home BP values, we further extracted pre-treatment home BP data from 68 patients (men: 39.7%, age: 69.5 years) who had not been treated and participated in the previous examination 4 years before the current examination.

### BP measurements

The home BP and office BPs collected in daily clinical practice were not considered in the present study. Instead, the home BP and office BP collected during the investigation of the Ohasama study was used for the analysis to maintain the consistency among participants.

Patients were asked to measure their BP at home for 4 weeks during the Ohasama study examinations. Study investigators or public health nurses instructed participants how to measure their home BP using the HEM7080IC cuff-oscillometric upper arm-cuff BP monitoring device (Omron Healthcare Co., Ltd., Kyoto, Japan) [22, 23]. The participants were instructed to measure their BP once each morning, rest for at least 2 min prior to measurement, within 1 h of waking up, with the cuff at heart level, and without taking any BP medication prior to measurement [24]. Evening BP was measured once each evening in the sitting position just before bedtime and after ≥2 min of rest [24]. To eliminate bias in individual selection, the initial measurement from each reading was used if the patients measured their BP twice or more on each occasion [24**–**27]. We defined home BP as the mean of all first readings on each occasion.

Office BP was measured by the study staff twice consecutively at each visit using the oscillometric Omron HEM-907IT device or HEM-9000AI (Omron Healthcare Co., Ltd.) [28]. Office BP was the average of these two readings measured in the study examinations, not at clinic visits in daily clinical practice, as we mentioned above. The participants were not asked to limit their use of antihypertensive medications before the examinations.

### Definition of BP control conditions

Controlled BP was defined as systolic BP < 135 mmHg and diastolic BP < 85 mmHg for home BP and <140/ < 90 mmHg for office BP [12, 29]. White-coat uncontrolled hypertension (WUCH) was defined as office systolic BP ≥ 140 mmHg or office diastolic BP ≥ 90 mmHg, but home systolic BP < 135 mmHg and home diastolic BP < 85 mmHg. Masked uncontrolled hypertension (MUCH) was defined as home systolic BP ≥ 135 mmHg or home diastolic BP ≥ 85 mmHg, but office systolic BP < 140 mmHg and office diastolic BP < 90 mmHg. Sustained uncontrolled hypertension (SUCH) was defined as an uncontrolled BP based on both office and home BP levels.

Following the Japanese Society of Hypertension 2019 guideline, we also defined home BP control conditions in a sensitivity analysis using home BP target values of <125/ < 75 mmHg in principle, but <135/ < 85 mmHg for patients without diabetes but with a history of CVD or age ≥75 years [1].

Controlled home BP conditions were evaluated using morning and evening home BP measurements.

### BP management in the HTC

In addition to the Ohasama study, the physicians of the Ohasama study group (MH, KA, KM, TO, and YI) treat hypertension in Ohasama residents based on home BP in the HTC. The HTC in this study refers to a hypertension outpatient clinic at the Iwate Prefectural Central Hospital Attachment Ohasama Regional Clinical Center (formerly Ohasama Hospital). We defined the HTC as a medical institute operated by physicians with extensive expertise in hypertension management, providing specialized care for patients with hypertension. In the present study, the physicians at the HTC were Fellows of the Japanese Society of Hypertension (YI, KA), a certified hypertension specialist (HM), associate editors or editorial board members of hypertension-related scientific journals (YI, TO, HM, KM, KA), or council members of the Japanese Society of Hypertension (TO, KA). Individuals newly diagnosed with hypertension during health check-ups, patients undergoing treatment but dissatisfied with their BP control, or those referred by other physicians for hypertension management generally visit the HTC.

Patients visiting the HTC were asked to measure their home BP with the home BP device (HEM7080IC or HEM-9700T [30]; Omron Healthcare Co., Ltd.) and to bring the monitor to each visit. The clinic staff member extracted home BP readings from the device using a computer and automatically calculated the averages and graphs of the patient’s home BP. Physicians at the HTC evaluated the home BP data and adjusted the medication to maintain home systolic/diastolic BP values < 135/ < 85 mmHg. Visits to the HTC were reviewed using medical records.

Non-HTC refers to clinics other than the HTC mentioned above. The method of hypertension management in non-HTCs was unknown; we could not collect data on whether patients regularly measured their home BP, whether home BP was used as a reference for BP assessment, and how physicians titrated anti-hypertensive treatments in non-HTCs.

### Other data

Drug information was collected by reviewing medical records and medication notes, which are widely distributed to patients in Japan. The classification of anti-hypertensive drugs is presented in Supplementary Table 1. Data on smoking and alcohol consumption habits, medical history, medications, weight, and height were obtained using self-reported questionnaires, which were distributed at the same time as the distribution of home BP measurement equipment. Since we started collecting information on weight and height in 2017, we also extracted data on weight and height from the examinations we conducted, if available.

### Statistical analysis

The means and proportions in the two groups were tested using Welch’s *t* test and Fisher’s exact test, respectively. The 95% confidence intervals of the proportions were calculated using the exact method. The proportion of patients with controlled office BP from the 2016 Japanese National Survey was used as one of the comparators [1, 8**]**.

Multivariate analysis was performed to explore the factors associated with controlled BP using the robust Poisson model, which can compute adjusted proportion ratios [31]. The dependent variable was home BP control, and three statistical models were applied. Model 1 included independent variables such as sex, age, body mass index (BMI), current smoking status, current alcohol consumption, diabetes, dyslipidemia, and history of cardiovascular disease (CVD). Model 2 incorporated the use of three or more classes of antihypertensive drugs in addition to Model 1, and Model 3 further included HTC visits. Missing BMI data (*n* = 68) and anti-hypertensive treatment regimens (*n* = 10) were imputed using multiple imputations by chained equations using the model with all independent variables and a dependent variable (with 100 datasets) [32].

All data were analyzed using SAS software (version 9.4 TS Level 1M8; SAS Institute, Cary, North Carolina, USA). Statistical significance was set at a *p* value of <0.05. Continuous variables are expressed as the mean ± standard deviation.

## Results

### Patient characteristics

Compared with the 207 patients who visited non-HTCs, the 172 patients who visited the HTC had a lower proportion of diabetes and lower home BP values. The patients in the HTC also had a higher proportion of patients receiving angiotensin II receptor blocker (ARB), thiazides (including thiazide-like diuretics), or mineralocorticoid receptor (MR) blockers, resulting in a higher number of anti-hypertensive classes being prescribed (Table 1).

**Table 1:** Patient characteristics

Characteristics	Non-HTCs (*n* = 207)	HTC (*n* = 172)	*p* value
Men, %	46.4	39.0	0.18
Age, years	71.5 ± 10.2	71.8 ± 8.6	0.73
Body mass index, kg/m^2^ (*n* = 154/157)*	25.0 ± 4.7	24.1 ± 3.0	0.055
Smoker, %	11.1	9.3	0.61
Drinker, %	50.7	52.3	0.76
Diabetes, %	21.7	12.2	0.020
Dyslipidemia, %	54.6	50.6	0.47
History of cardiovascular disease, %	13.5	10.5	0.43
Home morning BP, mmHg			
Systolic BP	138.2 ± 11.2	123.1 ± 7.5	<0.0001
Diastolic BP	78.3 ± 9.4	71.2 ± 7.3	<0.0001
Home evening BP, mmHg (*n* = 205/170)*			
Systolic BP	128.0 ± 12.2	113.3 ± 9.6	<0.0001
Diastolic BP	70.6 ± 8.9	63.9 ± 7.6	<0.0001
Unknown information on drugs, %	4.8	0.0	0.0025
Anti-hypertensive drugs, % (*n* = 197/172)*			
Dihydropyridine CCBs	78.7	77.9	0.90
Non-dihydropyridine CCBs	0.0	1.2	0.22
ARBs	64.5	86.6	<0.0001
ACE inhibitors	3.6	0.0	0.016
β-/αβ-blockers	12.2	10.5	0.63
α-blockers	3.6	0.6	0.072
Thiazides (-like)	9.1	30.2	<0.0001
Loop	1.0	0.6	>0.99
MR blockers	2.5	23.8	<0.0001
Anti-hypertensive drug class			<0.0001
One	42.6	23.8	
Two	42.1	34.3	
Three or more	15.2	41.9	

^*^Number of participants with information

*HTC* hypertension-specialized clinic, *BP* blood pressure, *CCB* calcium channel blocker, *ARB* angiotensin II receptor blocker, *ACEI* angiotensin-converting enzyme inhibitor, *MR* mineralocorticoid receptor

### BP control in the HTC and the non-HTCs

**Fig. 1:**
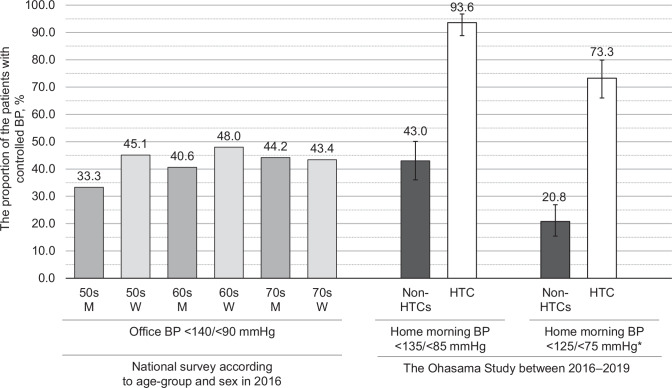
BP control in the national survey data and the present study. *Controlled home BP is defined as home morning BP < 125/ < 75 mmHg and <135/ < 85 mmHg for patients without diabetes but with a history of cardiovascular disease or age ≥75 years. Error bars indicate 95% confidence intervals. BP blood pressure, HTC hypertension-specialized clinic, M men, W women

As shown in Fig. 1, a higher proportion of patients with controlled home morning BP were observed in the HTC than in the non-HTCs. The proportion of patients with controlled home morning BP in the non-HTCs was similar to that of patients with controlled office BP in the 2016 national survey [1, 8]. The proportion of the patients with home morning BP below the Japanese Guidelines’ target [1] was 73.3% in the HTC, which was still higher than that in the non-HTCs. The proportion of patients with controlled home evening BP was 98.2% in the HTC and 78.1% in the non-HTCs (Supplementary Fig. 1), which was higher than the proportions of those with controlled home morning BP. The proportion of patients with both home morning and evening BP controlled to <135/ < 85 mmHg was 93.5% (95% confidence interval: 88.7–96.7%) in the HTC and 42.0% (95% confidence interval: 35.1–49.0%) in the non-HTC.

### Proportions of MUCH and WUCH

**Fig. 2:**
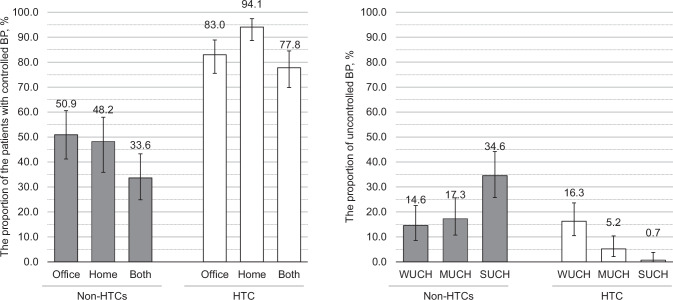
BP control based on both office and home morning BPs (*n* = 245). Controlled office and controlled home BP are defined as office BP < 140/ < 90 mmHg and home morning BP < 135/ < 85 mmHg, respectively (both BPs are measured during the study examinations and not during clinic visits). The analyses include 110 and 135 patients with office BP data from the non-HTC and HTC groups, respectively. Error bars indicate 95% confidence intervals. BP blood pressure, HTC hypertension-specialized clinic, WUCH white-coat uncontrolled hypertension, MUCH masked uncontrolled hypertension, SUCH sustained uncontrolled hypertension

Figure 2 shows the results when patients were classified according to their office and home morning BP values. The proportion of patients with controlled home morning BP was higher than that of patients with controlled office BP in the HTC. The proportion of patients with WUCH was higher than that of patients with MUCH or SUCH. The opposite phenomenon was observed in the non-HTCs. When home evening BP was used, the proportion of patients with WUCH was highest among the hypertension categories in both the non-HTC and HTC groups (Supplementary Fig. 2).

### Longitudinal change in BP

Among the 68 patients with available BP data between 2012 and 2015 (4 years before the current examination), the home morning BP levels in those not undergoing anti-hypertensive treatment were similar between the patients in the non-HTC (*n* = 16) and those in the HTC (*n* = 52). Meanwhile, the home morning and evening SBP and home evening DBP achieved by anti-hypertensive treatment was significantly lower in the HTC than in the non-HTCs (Table 2).

**Table 2:** Characteristics of patients not receiving anti-hypertensive medication at the visit 4 years before the current examination

Characteristics	Non-HTCs (*n* = 16)	HTC (*n* = 52)	*p* value
Men, %	37.5	40.4	>0.99
Age, years	71.6 ± 11.1	68.8 ± 9.6	0.37
Body mass index, kg/m^2^ (*n* = 16/44)*	22.9 ± 4.0	23.8 ± 3.3	0.43
Smoker, %	6.3	9.6	>0.99
Drinker, %	50.0	48.1	>0.99
Diabetes, %	25.0	9.6	0.20
Dyslipidemia, %	62.5	44.2	0.26
History of cardiovascular disease, %	12.5	3.8	0.23
4 years before the current examination (without anti-hypertensive treatment)			
Home morning SBP, mmHg	137.3 ± 11.1	142.1 ± 13.0	0.16
Home morning DBP, mmHg	76.4 ± 11.7	82.7 ± 10.6	0.066
Home evening SBP, mmHg (*n* = 16/51)*	129.4 ± 10.9	132.2 ± 17.3	0.45
Home evening DBP, mmHg (*n* = 16/51)*	69.7 ± 9.1	74.3 ± 11.1	0.10
Current examination (under anti-hypertensive treatment)			
Home morning SBP, mmHg	135.3 ± 11.1	122.4 ± 6.9	0.0003
Home morning DBP, mmHg	76.2 ± 10.7	70.6 ± 8.0	0.068
Home evening SBP, mmHg (*n* = 16/51)*	125.2 ± 7.4	112.7 ± 8.9	<0.0001
Home evening DBP, mmHg (*n* = 16/51)*	67.6 ± 8.1	62.8 ± 7.1	0.046
Anti-hypertensive drug class, % (*n* = 15/52)*			0.68
One	53.3	46.2	
Two	40.0	36.5	
Three or more	6.7	17.3	

^*^Number of participants with information

*SBP* systolic blood pressure, *DBP* diastolic blood pressure, *HTC* hypertension-specialized clinic

### Factors associated with controlled home BP

Table 3 shows the factors associated with controlled home morning BP among patient characteristics in the total of 379 patients. In Model 2, younger age, lower BMI, and use of ≥3 anti-hypertensive drug classes were associated with good home morning BP control. However, the association between ≥3 anti-hypertensive drug classes and controlled home morning BP was attenuated to a non-significant level after adding HTC visits to the model, suggesting that HTC visits are the stronger factor in controlled home morning BP than the number of anti-hypertensive drug classes. In the same analysis, after changing the dependent variable to home evening BP control, lower BMI and HTC visits were the only factors associated with good home evening BP control in Model 3 (Supplementary Table 2).

**Table 3:** Factors associated with controlled home morning BP

Associated factors	Proportion ratio of controlled home morning BP (95% confidence interval)		
	Model 1	Model 2	Model 3
Men ( = 1, Women=0)	0.91 (0.76–1.09)	0.88 (0.73–1.05)	0.98 (0.83–1.15)
Age (per 10 years)	0.92 (0.85–1.00)	0.91 (0.83–0.99) *	0.91 (0.84–0.99) *
Body mass index (per 5 kg/m^2^)	0.83 (0.73–0.94) *	0.82 (0.72–0.92) *	0.86 (0.77–0.96) *
Smoker ( = 1, non=0)	0.89 (0.67–1.18)	0.83 (0.63–1.10)	0.89 (0.70–1.13)
Drinker ( = 1, non=0)	1.03 (0.86–1.22)	1.03 (0.86–1.23)	0.96 (0.82–1.13)
Diabetes ( = 1, non=0)	0.92 (0.75–1.12)	0.96 (0.78–1.17)	1.03 (0.86–1.23)
Dyslipidemia ( = 1, non=0)	1.10 (0.95–1.27)	1.10 (0.95–1.27)	1.11 (0.98–1.26)
History of cardiovascular disease ( = 1, non=0)	0.95 (0.76–1.20)	0.94 (0.75–1.18)	0.99 (0.81–1.20)
Anti-hypertensive drugs ≥3 classes ( = 1, <3 classes=0)	[Not included]	1.39 (1.21–1.59) *	1.08 (0.96–1.21)
HTC visit ( = 1, non-HTCs=0)	[Not included]	[Not included]	2.10 (1.78–2.48) *

Model 1 simultaneously includes men, age, body mass index, smoking and drinking status, diabetes, dyslipidemia, and history of cardiovascular disease. Model 2 includes the use of ≥3 anti-hypertensive drug classes in addition to the variables in Model 1. Model 3 includes all the listed variables

*HTC* hypertension-specialized clinic, *BP* blood pressure

*p value < 0.05

## Discussion

The proportion of patients with controlled home BP was more than 90% in the HTC. Meanwhile, the proportion of patients with controlled home morning BP in the non-HTCs was approximately 40%; this proportion of patients with controlled home morning BP in the non-HTCs was equivalent to that of controlled office BP in the 2016 Japanese survey [1, 8]. When the patients were divided according to office and home morning BP status, the proportion of patients with MUCH or SUCH was lower in the HTC than in the non-HTCs.

The present results suggest that more than 90% of hypertensive patients can achieve home BP < 135/ < 85 mmHg in HTCs where management of hypertension is based on home BP measurements. The proportion of patients with controlled home morning BP among the non-HTCs was 43% and appeared not to be poor compared with other general clinics because a recent Japanese nationwide survey reported that the proportion of patients with controlled clinical hypertension (office BP < 140/ < 90 mmHg) was 33–48% [1, 8]. The proportion of patients with controlled home morning BP was 34% in the Japan Home versus Office Blood Pressure Measurement Evaluation (J-HOME) study conducted 20 years ago by general practitioners in Japan [33]. Compared with the data from the J-HOME study, the proportion of patients with controlled home BP in non-HTCs in this study is relatively high, suggesting that home BP control has improved over the past two decades [34, 35]. However, based on the results of this study, there is room for significant improvements in hypertension management.

A higher number of anti-hypertensive drug classes were prescribed to patients in the HTC than those in the non-HTCs. Among all the anti-hypertensive classes, ARBs, thiazides or thiazide-like diuretics, and MR blockers were prescribed more often in HTC than in non-HTCs. Similar to previous reports [36**–**38], ARBs and CCBs were frequently prescribed in the present study. However, it appears that one or more additional anti-hypertensive drug classes are needed as an option to achieve 90% of home BP control. Multiple regimens of different classes of anti-hypertensive drugs are effective for lowering BP [38**–**41]. Diuretics are recommended as one of the first-line anti-hypertensive regimens and β-blockers as a major anti-hypertensive drug [1]. Meanwhile, the HTC tended to prescribe more thiazides (or thiazide-like) diuretics and MR blockers, while the proportion of patients treated with β-/αβ-/α-blockers was similar in the HTC and non-HTCs. Combining MR blockers with ARBs and CCBs may be beneficial for lowering BP [42]. We previously estimated that increasing anti-hypertensive medication to ≥3 classes would improve 40% of uncontrolled BP cases in treated patients with hypertension [38].

Multivariate analysis implies that HTC visits per se may be a strong factor in controlling home BP. This finding is supported by a recent Japanese report indicating a significant association between consulting hypertension specialists and controlled office BP [43]. There are several possible explanations for this association. First, systematic treatment based on the patients’ home BP values may have prevented clinical inertia in hypertension management, resulting in good home BP control. The low proportion of patients with MUCH in the HTC group supported this hypothesis. Previous meta-analyses have suggested that anti-hypertensive treatment based on home BP monitoring can improve BP control [16, 44]. Second, regular home BP measurements and careful physician counseling based on a patient’s home BP data may have improved medication adherence and health awareness in the HTC. Third, physicians experienced in the treatment of hypertension can appropriately titrate anti-hypertensive drugs for each patient. A recent study suggested that personalized anti-hypertensive treatment has the potential to achieve an additional 4.4 mmHg lower systolic BP [45]. We previously reported that many general practitioners do not know the reference values for home BP [46, 47], suggesting that physician education on the appropriate use and evaluation of home BP is required to improve hypertension management.

In the present study, home evening BP was controlled more effectively than home morning BP. This may be because the Japanese generally have the habit of bathing and drinking in the evening. However, it should be noted that home morning BP can be a good predictor of cardiovascular disease, especially among treated patients [15, 48].

### Limitations

This study had some limitations. First, the HTC may have included patients who were seriously concerned about hypertension. It is possible that health consciousness about hypertension was the cause of improved BP control at home. Furthermore, we used data from patients who participated in the primary part of the Ohasama study. The study population consisted of patients who were able to keep visiting regular outpatient clinics. Therefore, healthy participant bias might have existed in the present study. Second, the Japanese Society of Hypertension released new hypertension guidelines in April 2019, which lowered the target BP by 10 mmHg in most cases [1]. Home BP control may have improved after this update for non-HTCs. However, a previous survey based on large-scale Japanese data suggested that office BP was not significantly altered in 2019 [20]. A recent Japanese cross-sectional study found that only approximately 20% of patients achieved home BP levels below 125/75 mmHg [49]. Third, pre-treatment BP was unknown for most patients in the present study. Furthermore, we could not quantify the extent to which lifestyle modifications such as sodium reduction, physical activity, and maintenance of appropriate body weight were made in the HTC due to the lack of information. However, the result after adjustment for BMI partly suggests that visiting the HTC per se may have contributed to controlled home BP beyond lifestyle modifications because BMI reflects unpreferable lifestyles. Finally, information regarding drug adherence was not collected. Improvements in drug adherence are crucial for controlling BP.

### Perspective of Asia

Improving BP control remains a challenge in Asia [12]. The success of HTC in achieving excellent BP control rates could provide valuable insights for Asian countries seeking to enhance their hypertension management strategies. Implementing the strict BP control based on the self-measurement at home, as demonstrated in this study, may offer a solution to this persistent issue. The advancement of digital health technologies, including digital hypertension strategies, may potentially contribute to addressing the ongoing challenges in hypertension management [50]. Future research should explore how to expand the strict BP management based on home BP monitoring in clinical practice.

## Conclusion

In conclusion, the proportion of patients with home BP control was excellent in the HTC. Although a high number of anti-hypertensive drug classes was associated with good home BP control, the multivariable analysis showed that the HTC visit, in which patients’ BP was managed based on home BP per se, may have been a strong factor in BP control. Strict home BP control reduces the risk of cardiovascular disease in the Japanese patient population [7, 51]. We have confirmed that the incidence of stroke has decreased in Ohasama town [52]. Home BP-based hypertension practices, as recommended in the current Japanese guidelines, may be the key to achieving sufficient BP control and preventing cardiovascular disease.

## Supplementary information

Supplemental Materials

## References

[1] Umemura S, Arima H, Arima S, Asayama K, Dohi Y, Hirooka Y, et al. The Japanese Society of Hypertension Guidelines for the Management of Hypertension (JSH 2019). Hypertens Res. 2019;42:1235–481. 31375757 10.1038/s41440-019-0284-9

[2] Whelton PK, Carey RM, Aronow WS, Casey DE Jr, Collins KJ, Dennison Himmelfarb C, et al. 2017 ACC/AHA/AAPA/ABC/ACPM/AGS/APhA/ASH/ASPC/NMA/PCNA Guideline for the Prevention, Detection, Evaluation, and Management of High Blood Pressure in Adults: A Report of the American College of Cardiology/American Heart Association Task Force on Clinical Practice Guidelines. Hypertension. 2018;71:e13–e115. 29133356 10.1161/HYP.0000000000000065

[3] Mancia G, Kreutz R, Brunstrom M, Burnier M, Grassi G, Januszewicz A, et al. 2023 ESH Guidelines for the management of arterial hypertension The Task Force for the management of arterial hypertension of the European Society of Hypertension: Endorsed by the International Society of Hypertension (ISH) and the European Renal Association (ERA). J Hypertens. 2023;41:1874–2071. 37345492 10.1097/HJH.0000000000003480

[4] Satoh M, Asayama K, Kikuya M, Inoue R, Metoki H, Hosaka M, et al. Long-term stroke risk due to partial white-coat or masked hypertension based on home and ambulatory blood pressure measurements: the Ohasama study. Hypertension. 2016;67:48–55. 26527046 10.1161/HYPERTENSIONAHA.115.06461

[5] SPRINT Research Group, Lewis CE, Fine LJ, Beddhu S, Cheung AK, Cushman WC, et al. Final report of a trial of intensive versus standard blood-pressure control. N Engl J Med. 2021;384:1921–30. 34010531 10.1056/NEJMoa1901281 PMC9907774

[6] Zhang W, Zhang S, Deng Y, Wu S, Ren J, Sun G, et al. Trial of intensive blood-pressure control in older patients with hypertension. N Engl J Med. 2021;385:1268–79. 34491661 10.1056/NEJMoa2111437

[7] Asayama K, Ohkubo T, Metoki H, Obara T, Inoue R, Kikuya M, et al. Cardiovascular outcomes in the first trial of antihypertensive therapy guided by self-measured home blood pressure. Hypertens Res. 2012;35:1102–10. 22895063 10.1038/hr.2012.125

[8] Hisamatsu T, Segawa H, Kadota A, Ohkubo T, Arima H, Miura K. Epidemiology of hypertension in Japan: beyond the new 2019 Japanese guidelines. Hypertens Res. 2020;43:1344–51. 32636526 10.1038/s41440-020-0508-z

[9] Ohno K, Takase H, Sugiura T, Machii M, Nonaka D, Tokumaru M, et al. Current status and recent changes in blood pressure and dietary salt consumption in Japanese individuals. Clin Exp Hypertens. 2021;43:287–94. 33356624 10.1080/10641963.2020.1867158

[10] Fujishima S, Kodama S, Tsuchihashi T. Achievement rate of blood pressure <140/90 mmHg and <130/80 mmHg in subjects with hypertension; findings from a Japanese health checkup in 2017. Clin Exp Hypertens. 2020;42:648–55. 32419520 10.1080/10641963.2020.1764017

[11] Yokokawa H, Suzuki M, Aoki N, Sato Y, Naito T. Achievement of target blood pressure among community residents with hypertension and factors associated with therapeutic failure in the northern territory of Japan. J Int Med Res. 2022;50:3000605221126878. 36314244 10.1177/03000605221126878 PMC9623383

[12] N C D Risk Factor Collaboration. Worldwide trends in hypertension prevalence and progress in treatment and control from 1990 to 2019: a pooled analysis of 1201 population-representative studies with 104 million participants. Lancet. 2021;398:957–80. 34450083 10.1016/S0140-6736(21)01330-1 PMC8446938

[13] Phillips LS, Branch WT, Cook CB, Doyle JP, El-Kebbi IM, Gallina DL, et al. Clinical inertia. Ann Intern Med. 2001;135:825–34. 11694107 10.7326/0003-4819-135-9-200111060-00012

[14] Satoh M, Hirose T, Nakayama S, Murakami T, Takabatake K, Asayama K, et al. Blood pressure and chronic kidney disease stratified by gender and the use of antihypertensive drugs. J Am Heart Assoc. 2020;9:e015592. 32794421 10.1161/JAHA.119.015592 PMC7660816

[15] Asayama K, Ohkubo T, Hanazawa T, Watabe D, Hosaka M, Satoh M, et al. Does antihypertensive drug class affect day-to-day variability of self-measured home blood pressure? the HOMED-BP study. J Am Heart Assoc. 2016;5:e002995. 27009620 10.1161/JAHA.115.002995 PMC4943272

[16] Satoh M, Maeda T, Hoshide S, Ohkubo T. Is antihypertensive treatment based on home blood pressure recommended rather than that based on office blood pressure in adults with essential hypertension? (meta-analysis). Hypertens Res. 2019;42:807–16. 30948837 10.1038/s41440-019-0221-y

[17] Asayama K, Brguljan-Hitij J, Imai Y. Out-of-office blood pressure improves risk stratification in normotension and prehypertension people. Curr Hypertens Rep. 2014;16:478. 25139777 10.1007/s11906-014-0478-0

[18] Imai Y, Satoh H, Nagai K, Sakuma M, Sakuma H, Minami N, et al. Characteristics of a community-based distribution of home blood pressure in Ohasama in northern Japan. J Hypertens. 1993;11:1441–9. 8133026 10.1097/00004872-199312000-00017

[19] Satoh M, Yoshida T, Metoki H, Murakami T, Tatsumi Y, Hirose T, et al. The long-term reproducibility of the white-coat effect on blood pressure as a continuous variable from the Ohasama Study. Sci Rep. 2023;13:4985. 36973366 10.1038/s41598-023-31861-9 PMC10043024

[20] Satoh M, Metoki H, Asayama K, Murakami T, Inoue R, Tsubota-Utsugi M, et al. Age-related trends in home blood pressure, home pulse rate, and day-to-day blood pressure and pulse rate variability based on longitudinal cohort data: the Ohasama study. J Am Heart Assoc. 2019;8:e012121. 31333055 10.1161/JAHA.119.012121 PMC6761623

[21] Ohkubo T, Satoh M. Prognostic significance of home and ambulatory blood pressure: summary of longitudinal evidence from the Ohasama study. Tohoku J Exp Med. 2023;260:273–82. 37286522 10.1620/tjem.2023.J045

[22] Chonan K, Kikuya M, Araki T, Fujiwara T, Suzuki M, Michimata M, et al. Device for the self-measurement of blood pressure that can monitor blood pressure during sleep. Blood Press Monit. 2001;6:203–5. 11805470 10.1097/00126097-200108000-00008

[23] El Assaad MA, Topouchian JA, Asmar RG. Evaluation of two devices for self-measurement of blood pressure according to the international protocol: the Omron M5-I and the Omron 705IT. Blood Press Monit. 2003;8:127–33. 12900590 10.1097/00126097-200306000-00006

[24] Imai Y, Kario K, Shimada K, Kawano Y, Hasebe N, Matsuura H, et al. The Japanese society of hypertension guidelines for self-monitoring of blood pressure at home (second edition). Hypertens Res. 2012;35:777–95. 22863910 10.1038/hr.2012.56

[25] Kikuya M, Ohkubo T, Metoki H, Asayama K, Hara A, Obara T, et al. Day-by-day variability of blood pressure and heart rate at home as a novel predictor of prognosis: the Ohasama study. Hypertension. 2008;52:1045–50. 18981332 10.1161/HYPERTENSIONAHA.107.104620

[26] Asayama K, Kikuya M, Schutte R, Thijs L, Hosaka M, Satoh M, et al. Home blood pressure variability as cardiovascular risk factor in the population of Ohasama. Hypertension. 2013;61:61–9. 23172933 10.1161/HYPERTENSIONAHA.111.00138 PMC3607332

[27] Satoh M, Hosaka M, Asayama K, Kikuya M, Inoue R, Metoki H, et al. Association between N-terminal pro B-type natriuretic peptide and day-to-day blood pressure and heart rate variability in a general population: the Ohasama study. J Hypertens. 2015;33:1536–41. 25827428 10.1097/HJH.0000000000000570

[28] White WB, Anwar YA. Evaluation of the overall efficacy of the Omron office digital blood pressure HEM-907 monitor in adults. Blood Press Monit. 2001;6:107–10. 11433132 10.1097/00126097-200104000-00007

[29] Shimamoto K, Ando K, Fujita T, Hasebe N, Higaki J, Horiuchi M, et al. The Japanese society of hypertension guidelines for the management of hypertension (JSH 2014). Hypertens Res. 2014;37:253–390. 24705419 10.1038/hr.2014.20

[30] Kuwabara M, Harada K, Hishiki Y, Kario K. Validation of an automatic device for the self-measurement of blood pressure in sitting and supine positions according to the ANSI/AAMI/ISO81060-2: 2013 guidelines: the Omron HEM-9700T. Blood Press Monit. 2019;24:146–50. 31026232 10.1097/MBP.0000000000000368

[31] Barros AJ, Hirakata VN. Alternatives for logistic regression in cross-sectional studies: an empirical comparison of models that directly estimate the prevalence ratio. BMC Med Res Methodol. 2003;3:21. 14567763 10.1186/1471-2288-3-21 PMC521200

[32] Moons KG, Donders RA, Stijnen T, Harrell FE Jr. Using the outcome for imputation of missing predictor values was preferred. J Clin Epidemiol. 2006;59:1092–101. 16980150 10.1016/j.jclinepi.2006.01.009

[33] Obara T, Ohkubo T, Funahashi J, Kikuya M, Asayama K, Metoki H, et al. Isolated uncontrolled hypertension at home and in the office among treated hypertensive patients from the J-HOME study. J Hypertens. 2005;23:1653–60. 16093909 10.1097/01.hjh.0000178334.33352.56

[34] Kario K, Tomitani N, Buranakitjaroen P, Chia YC, Park S, Chen CH, et al. Home blood pressure control status in 2017-2018 for hypertension specialist centers in Asia: Results of the Asia BP@Home study. J Clin Hypertens (Greenwich). 2018;20:1686–95. 30444315 10.1111/jch.13415 PMC8031357

[35] Kario K, Hoshide S, Tomitani N, Nishizawa M, Yoshida T, Kabutoya T, et al. Inconsistent control status of office, home, and ambulatory blood pressure all taken using the same device: the HI-JAMP study baseline data. Am J Hypertens. 2023;36:90–101. 36053278 10.1093/ajh/hpac103

[36] Ohishi M, Yoshida T, Oh A, Hiroi S, Takeshima T, Otsuka Y, et al. Analysis of antihypertensive treatment using real-world Japanese data-the retrospective study of antihypertensives for lowering blood pressure (REAL) study. Hypertens Res. 2019;42:1057–67. 30842611 10.1038/s41440-019-0238-2 PMC8075880

[37] Obara T, Ito K, Ohkubo T, Shibamiya T, Shinki T, Nakashita M, et al. Uncontrolled hypertension based on morning and evening home blood pressure measurements from the J-HOME study. Hypertens Res. 2009;32:1072–8. 19779486 10.1038/hr.2009.152

[38] Satoh M, Muroya T, Murakami T, Obara T, Asayama K, Ohkubo T, et al. The impact of clinical inertia on uncontrolled blood pressure in treated hypertension: real-world, longitudinal data from Japan. Hypertens Res. 2024;47:598–607. 37872377 10.1038/s41440-023-01452-2

[39] Wald DS, Law M, Morris JK, Bestwick JP, Wald NJ. Combination therapy versus monotherapy in reducing blood pressure: meta-analysis on 11,000 participants from 42 trials. Am J Med. 2009;122:290–300. 19272490 10.1016/j.amjmed.2008.09.038

[40] Hosaka M, Metoki H, Satoh M, Ohkubo T, Asayama K, Kikuya M, et al. Randomized trial comparing the velocities of the antihypertensive effects on home blood pressure of candesartan and candesartan with hydrochlorothiazide. Hypertens Res. 2015;38:701–7. 26041602 10.1038/hr.2015.64

[41] Metoki H, Ohkubo T, Kikuya M, Asayama K, Inoue R, Obara T, et al. The velocity of antihypertensive effect of losartan/hydrochlorothiazide and angiotensin II receptor blocker. J Hypertens. 2012;30:1478–86. 22573119 10.1097/HJH.0b013e328353f1fe

[42] Elnagar N, Satoh M, Hosaka M, Asayama K, Ishikura K, Obara T, et al. The velocity of home blood pressure reduction in response to low-dose eplerenone combined with other antihypertensive drugs determined by exponential decay function analysis. Clin Exp Hypertens. 2014;36:83–91. 24625334 10.3109/10641963.2014.892117

[43] Sakima A, Yamazato M, Kohagura K, Ishida A, Matayoshi T, Tana T, et al. Achievement rate of target blood pressure in patients with hypertension treated by hypertension specialists and non-specialists in a real-world setting. Hypertens Res. 2023;46:2460–9. 37414873 10.1038/s41440-023-01362-3

[44] Tucker KL, Sheppard JP, Stevens R, Bosworth HB, Bove A, Bray EP, et al. Self-monitoring of blood pressure in hypertension: a systematic review and individual patient data meta-analysis. PLoS Med. 2017;14:e1002389. 28926573 10.1371/journal.pmed.1002389 PMC5604965

[45] Sundstrom J, Lind L, Nowrouzi S, Hagstrom E, Held C, Lytsy P, et al. Heterogeneity in blood pressure response to 4 antihypertensive drugs: a randomized clinical trial. JAMA. 2023;329:1160–9. 37039792 10.1001/jama.2023.3322 PMC10091169

[46] Obara T, Ohkubo T, Fukunaga H, Kobayashi M, Satoh M, Metoki H, et al. Practice and awareness of physicians regarding home blood pressure measurement in Japan. Hypertens Res. 2010;33:428–34. 20186152 10.1038/hr.2010.10

[47] Wang TD, Ohkubo T, Bunyi ML, Chadachan VM, Chia YC, Kario K, et al. Current realities of home blood pressure monitoring from physicians’ perspectives: results from Asia HBPM survey 2020. Hypertens Res. 2023;46:1638–49. 37041412 10.1038/s41440-023-01259-1 PMC10319632

[48] Asayama K, Ohkubo T, Kikuya M, Obara T, Metoki H, Inoue R, et al. Prediction of stroke by home “morning” versus “evening” blood pressure values: the Ohasama study. Hypertension. 2006;48:737–43. 16952977 10.1161/01.HYP.0000240332.01877.11

[49] Kobayashi K, Chin K, Hatori N, Furuki T, Sakai H, Miyakawa M, et al. Cross-sectional survey of hypertension management in clinical practice in Japan: the Kanagawa Hypertension Study 2021 conducted in collaboration with Japan Medical Association Database of Clinical Medicine. Hypertens Res. 2023;46:2447–59. 37532949 10.1038/s41440-023-01366-z

[50] Kario K, Williams B, Tomitani N, McManus RJ, Schutte AE, Avolio A, et al. Innovations in blood pressure measurement and reporting technology: International Society of Hypertension position paper endorsed by the World Hypertension League, European Society of Hypertension, Asian Pacific Society of Hypertension, and Latin American Society of Hypertension. J Hypertens. 2024;42:1874–88. 10.1097/HJH.0000000000003827 39246139

[51] Kario K, Saito I, Kushiro T, Teramukai S, Tomono Y, Okuda Y, et al. Morning home blood pressure is a strong predictor of coronary artery disease: the HONEST study. J Am Coll Cardiol. 2016;67:1519–27. 27150682 10.1016/j.jacc.2016.01.037

[52] Imai Y. A personal history of research on hypertension From an encounter with hypertension to the development of hypertension practice based on out-of-clinic blood pressure measurements. Hypertens Res. 2022;45:1726–42. 36075990 10.1038/s41440-022-01011-1 PMC9637554

