# Supplementary material for: Home blood pressure control and prescribing patterns of anti-hypertensive medications in a home blood pressure-based hypertension-specialized clinic in Japan: a sub-analysis of the Ohasama study
Source: Hypertens Res. 2024 Oct 28;48(1):26–36. doi: 10.1038/s41440-024-01954-7 (PMC11700850; doi:10.1038/s41440-024-01954-7)
Supplement: Supplementary file 1 — Supplemental Materials [file 41440_2024_1954_MOESM1_ESM.pdf]

Supplemental Materials

Home blood pressure control and prescribing pattern of anti-hypertensive medications in home blood pressure-based hypertension-specialized clinic

Supplementary Table 1. Anti-hypertensive drug classification according to World Health Organization-ATC codes

Class	ATC Code
Dihydropyridine CCB	C08CA
Benzothiazepine CCB	C08DB
ARB	C09CA
ACEI	C09AA
Thiazide diuretic<br>(including thiazide-like diuretic)	C03AA, C03BA
β-blocker or<br>α-, β-blocker	C07AA, C07AB, C07AG
α-blocker	C02CA, G04CA03
MR antagonists<br>(aldosterone antagonists)	C03DA
Loop diuretic	C03CA010
Others	C02AA, C02AB, C02AC, C02DB, C03DB, C09XA

ARB, angiotensin II receptor blocker; ACEI, angiotensin-converting enzyme inhibitor; ATC, anatomical therapeutic chemical; CCB, calcium channel blocker; MR, mineralocorticoid receptor.

Supplementary Table 2. Factors associated with controlled home evening BP

	Proportion ratio of controlled home evening BP (95% confidence interval)		
**Demographic and health variables**	**Model 1**	**Model 2**	**Model 3**
Men (=1, Women=0)	1.02 (0.93–1.12)	1.01 (0.92–1.11)	1.05 (0.95–1.15)
Age (per 10 year)	0.99 (0.94–1.04)	0.99 (0.94–1.04)	0.99 (0.94–1.04)
Body mass index (per 5 kg/m^2)	0.89 (0.82–0.96) *	0.89 (0.82–0.96) *	0.90 (0.83–0.97) *
Smoker (=1, non=0)	0.93 (0.81–1.07)	0.92 (0.80–1.06)	0.94 (0.82–1.07)
Drinker (=1, non=0)	1.08 (0.98–1.19)	1.08 (0.98–1.19)	1.06 (0.96–1.16)
Diabetes (=1, non=0)	1.01 (0.90–1.12)	1.02 (0.91–1.13)	1.04 (0.94–1.15)
Dyslipidemia (=1, non=0)	1.05 (0.97–1.14)	1.05 (0.97–1.14)	1.05 (0.98–1.13)
History of cardiovascular disease (=1, non=0)	0.98 (0.87–1.11)	0.98 (0.87–1.11)	1.00 (0.89–1.12)
Anti-hypertensive drugs ≥3 classes (=1, <3 classes=0)	[Not included]	1.07 (0.99–1.16)	0.99 (0.91–1.07)
HTC visit (=1, non-HTCs=0)	[Not included]	[Not included]	1.26 (1.16–1.36) *

Model 1 simultaneously includes men, age, body mass index, smoking and drinking status, diabetes, dyslipidemia, and history of cardiovascular disease. Model 2 includes the use of anti-hypertensive drugs in addition to the variables in Model 1. Model 3 includes all the listed variables.
HTC, hypertension-specialized clinic; BP, blood pressure.
*p value <0.05

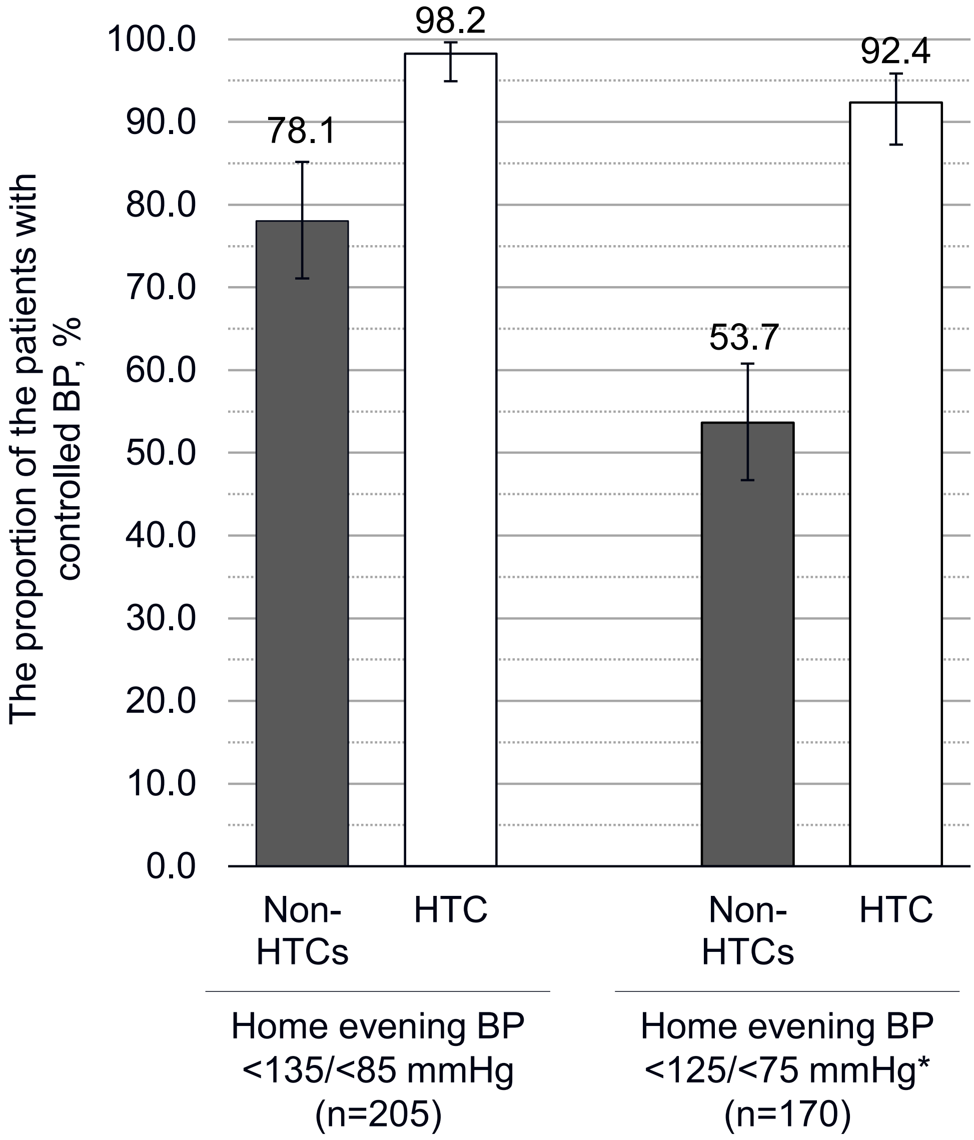

Supplementary Figure 1. BP control based on home evening BP (n=375)

Home evening BP is used instead of morning values after excluding 4 patients without home evening BP data. Error bars indicate 95% confidence intervals.

*Home BP control is defined as home evening BP <125/<75 mmHg but <135/<85 mmHg for patients without diabetes but with a history of cardiovascular disease or an age ≥75 years.

BP, blood pressure; HTC, hypertension-specialized clinic.

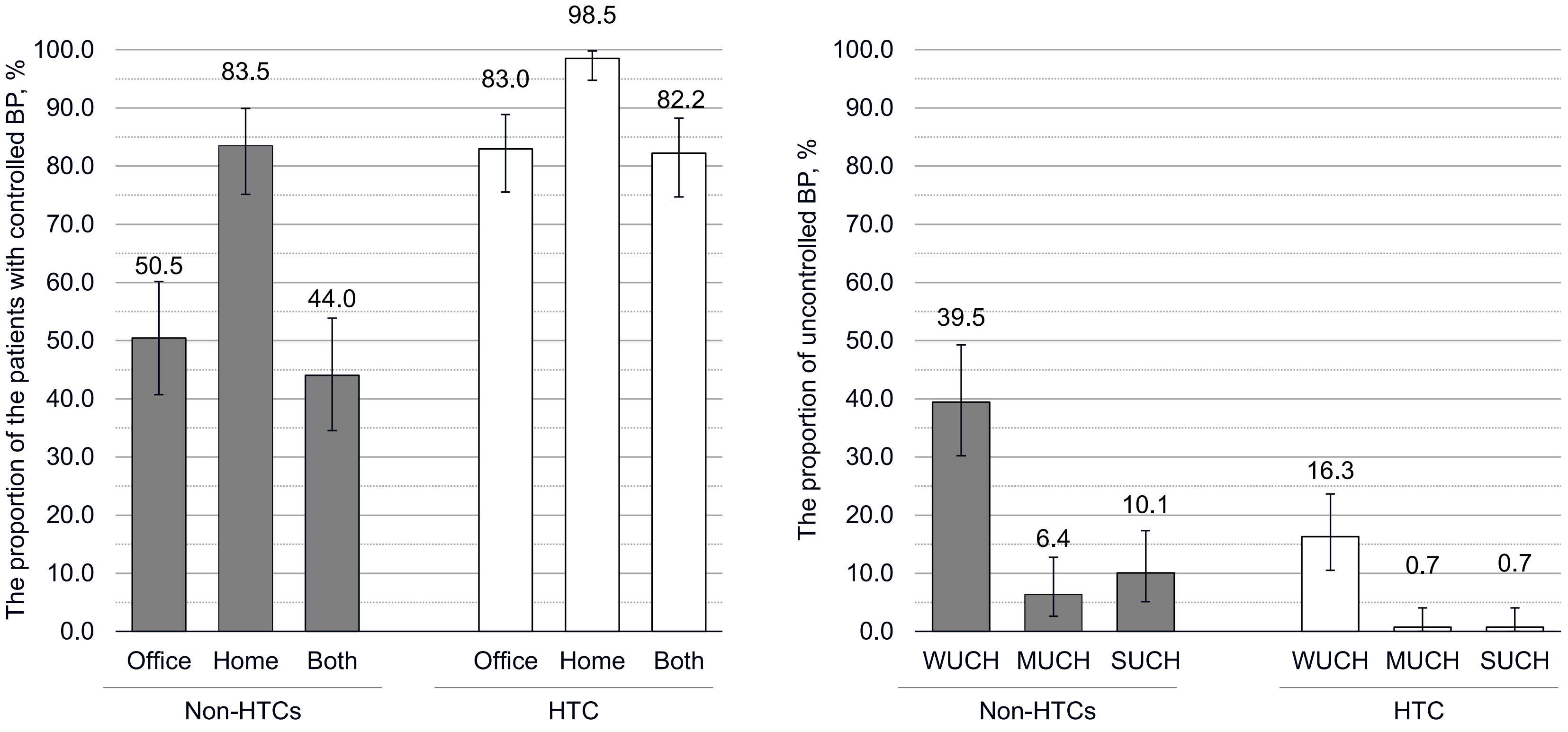

Supplementary Figure 2. BP control based on both office and home evening BPs (n=244)

Office and home evening BP control are defined as office BP <140/<90 mmHg and home evening BP <135/<85 mmHg, respectively (both BPs are measured during the study examinations and not during clinic visits). The analyses include 109 and 135 patients with office BP data from the non-HTCs and the HTC, respectively.

Error bars indicate 95% confidence intervals.

BP, blood pressure; HTC, hypertension-specialized clinic; WUCH, white coat uncontrolled hypertension; MUCH, masked uncontrolled hypertension; SUCH, sustained uncontrolled hypertension.
